# Evaluation of conservation status of plants in Brazil’s Atlantic forest: An ethnoecological approach with *Quilombola* communities in Serra do Mar State Park

**DOI:** 10.1371/journal.pone.0238914

**Published:** 2020-09-18

**Authors:** Bruno Esteves Conde, Sonia Aragaki, Tamara Ticktin, Amanda Surerus Fonseca, Priscila Baptistella Yazbek, Thamara Sauini, Eliana Rodrigues

**Affiliations:** 1 Departament of Biological Sciences, Postgraduate Program in Chemical Biology, Universidade Federal de São Paulo, Diadema, São Paulo, Brazil; 2 Centro Universitário Estácio de Sá, Juiz de Fora, Minas Gerais, Brazil; 3 Instituto de Botânica de São Paulo, São Paulo, Brazil; 4 Botany Department, University of Hawaii at Manoa, Honolulu, Hawaii, United States of America; 5 Centro de Ensino Superior de Juiz de Fora, Juiz de Fora, Minas Gerais, Brazil; 6 Department of Environmental Sciences, Center for Ethnobotanical and Ethnopharmacological Studies, Universidade Federal de São Paulo, São Paulo, Brazil; 7 Department of Environmental Sciences, Universidade Federal de São Paulo, Diadema, São Paulo, Brazil; University of South Carolina, UNITED STATES

## Abstract

The Atlantic Forest is considered the fourth most important biodiversity hotspot. Although almost 96% of its original area has been devastated, a large part of its remaining conserved area is inhabited by traditional communities. This research focused on two *Quilombola* communities who reside within the Núcleo Picinguaba of the Serra do Mar State Park, State of São Paulo, Brazil. The objective was to use a combination of ethnoecological and ecological approaches to select priority species for which to develop participatory conservation and sustainable management plans in protected areas in Brazil. We collaborated with community members to collect ethnobotanical and ethnoecological data and then measured the abundance of native species in local forests through phytosociological sampling. We used this information to assess the degree of threat to useful species using the Conservation Priority Index, adding an additional layer of analysis based on habitat successional categories. We then overlayed those useful species identified as highest risk locally with those federally listed as threatened or endangered. Based on this, we identified three species as priority for the development of sustainable management plans: *Virola bicuhyba*, *Cedrella fissilis* and *Plinia edulis*.

## Introduction

Areas rich in biodiversity, with a large number of endemic species and which have a high degree of environmental degradation, were conceptualized by Myers [1] as a biodiversity hotspots. He thus mapped the priority areas of the planet for initiatives aimed at conservation.

Among these areas is the Atlantic Forest, the fourth most important hotspot among the 25 considered [2]. This biome had had almost 96% of its original area devastated [3], and its conservation is considered a challenge due to its high degree of disturbance, and that much of its remaining preserved area is inhabited by traditional communities [4].

According to Brazil’s constitutional decree No. 6,040 of February 7, 2007, traditional communities are culturally differentiated human groups that recognize themselves as such, and who occupy and use territories and natural resources as a condition for their cultural, religious and cultural reproduction. In Brazil, among the traditional communities, are the *Quilombolas* [4]. The *Quilombolas* are descendants of slaves of African origin who came to Brazil during the colonial (1530±1815), nited kingdom (1815±1822) and empire (1822±1889) periods. Some of these slaves fled the farms on which they were exploited, organizing communities of refugees called *Quilombolas*, in the local forests. Since that time, the *Quilombolas* have lived in villages where they have made a living from agriculture and use of forest resources [5]. According to Peralta [6], to date there is no certainty about how many Quilombola communities there are, however, data from the Brazilian Government estimates that there are about 3,000 Q*uilombola* Communities in Brazil, with approximately 100 *Quilombola* communities in the Atlantic Forest [7]. Since these communities use the local flora as a means of meeting their basic demands for survival, it is essential that local use and conservation are compatible. *Quilombola* communities have lived and interacted with forests for a long time, developing detailed “traditional ecological knowledge” (TEK) [8]. TEK is developed through the process of observation and experimentation, transmitted among individuals and across generations [9] and is integral to the development of conservation and management plans in traditional communities today. The involvement and active participation of local residents is fundamental for the co-management, production, use and management of plant biodiversity resources [10]. Local and participatory management integrates local culture and knowledge and conservation [11].

There is a small but growing literature on traditional use of resources and biodiversity conservation in *Quilombola* communities. Hoffman [12] studied the impact of use on forest plants by a *Quilombola* community in Suriname. Austin-Ragosta [13] studied historical influences on the development of Jamaican *Quilombola* knowledge and biodiversity conservation, focusing on ethnomedicine. In Brazil, few studies have assessed traditional knowledge and biodiversity conservation in *Quilombola* communities. However, Crepaldi, Peixoto [14] and Conde and collaborators [5] evaluated the potential for sustainable harvest of plant resources based on traditional knowledge and species abundance in different *Quilombola* communities. Beyond *Quilombola* communities, many studies have used a combination of ecological and ethnographic approaches to assess sustainable resource use in local and indigenous communities [15, 16]. The Conservation Priority Index (IPC) is often used as a methodology for these assessments, especially in the context of traditional communities who use forest resources to meet many of their subsistence needs. This index assesses the conservation status of locally important plant resources by combing information on the local abundance of species in their natural environments, with the risk they face based on the method of harvest and the frequency and types of uses. Here, we adapt this method to include an additional consideration–the ecological successional habitat of the species.

The objective of this study was to use a combination of ethnobotanical and ecological approaches to select priority species for the development of participatory resource management plans in a protected area—Núcleo Picinguaba of the Serra do Mar State Park, State of São Paulo, Brazil. The broader goal is to foster the conservation and sustainable use of plant species in this region.

## Methodology

### Study area

**Fig 1:**
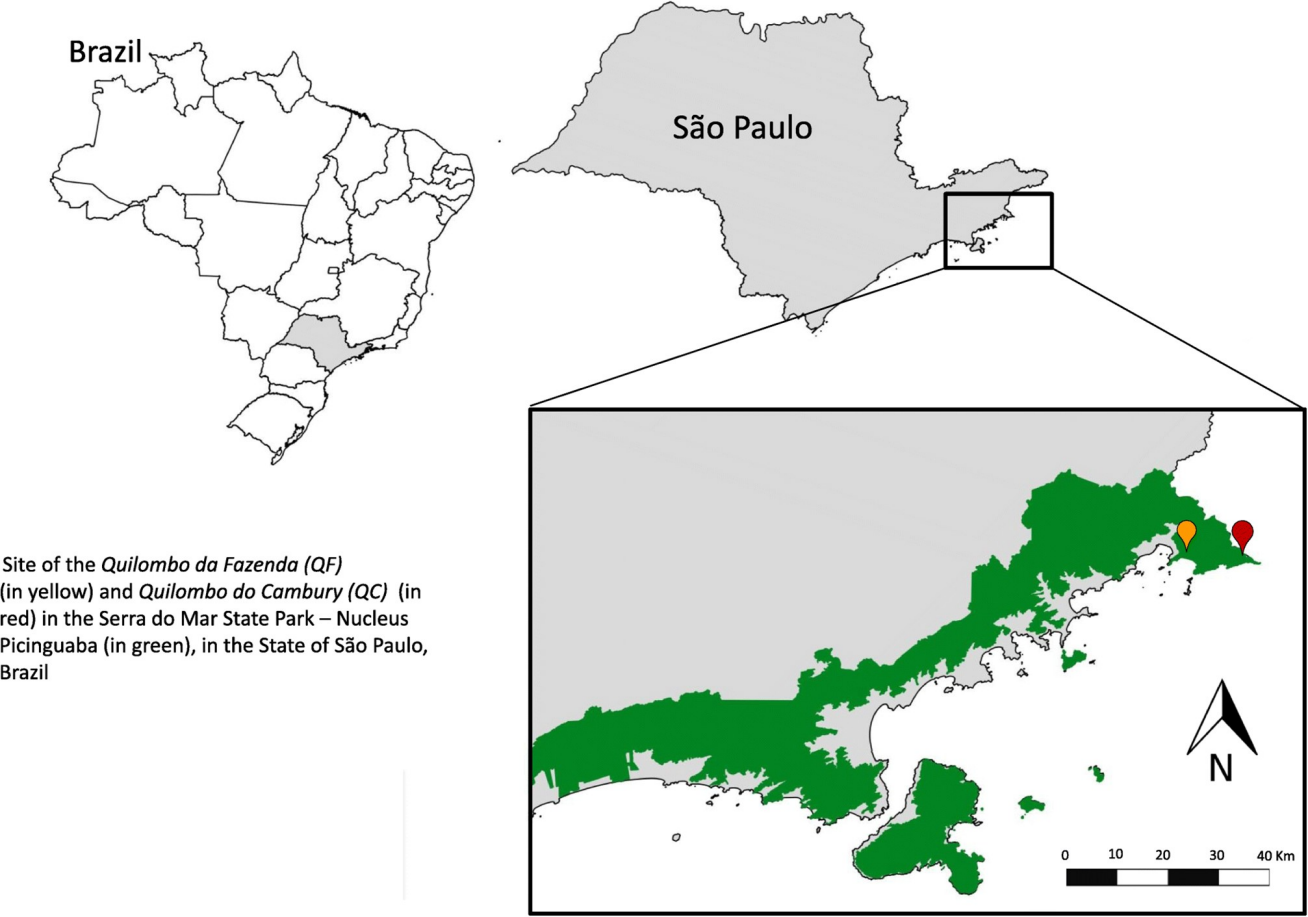
Site of the *Quilombo da Fazenda* (QF) (*in yellow*) and *Quilombo do Cambury* (QC) (in red) in the Serra do Mar State Park–Nucleus Picinguaba (in green), in the State of São Paulo, Brazil.

Our research focused on two *Quilombola* communities (Fig 1), certified by Fundação Cultural Palmares since 2005 [17]. The first is Quilombo da Fazenda (QF), which dates back to the end of the 19th century and today consists of about 40 families (170 people). It overlaps with the protected area—the Núcleo Picinguaba of the Serra do Mar State Park, which represents the largest conservation park and portion of continuous conservation of the Atlantic Forest in Brazil. The second is Quilombo do Cambury (QC), which dates back more than 150 years and today has approximately 50 families (230 people). QC is locatedin the Serra da Bocaina Mosaic, in the north of São Paulo and Sul Fluminense, forming a significant ecological corridor for the protection of the Atlantic Forest [18]. Livelihoods in these communities center on subsistence agriculture and the use of forest resources.

### Ethical aspects of research

Prior to data collection, all necessary legal licenses, as well as the participants’ consent to the use of the right to images, were obtained for the development of this study, as follows: 1) COTEC—Technical and Scientific Committee of Instituto Florestal, n°. 260108–009.510 / 2015for access to the Serra do Mar State Park area; 2) SISBIO—Biodiversity Information and Authorization System, n ° 51199–1 / 2015, for collecting and accessing plants in the Serra da Bocaina National Park; 3) SISGEN—National System for the Management of Genetic Heritage and Associated Traditional Knowledge, n. A648D14 to obtain prior informed consent and permission to inquire about traditional ecological knowledge; and 4) Research Ethics Committee No. 028525/2016 for the study to be carried out at the Federal University of São Paulo.

### Project genesis (2015)

This project involves the collaboration of members of the two communities (QF and QC)—including 5 community partners, who actively participated in all phases of the project (from genesis and data analysis to publication), 19 interviewees who participated directly in the project, and 40 others who participated indirectly during the filming, workshops, assemblies and other activities developed with the communities, as well as a team of researchers with experience in agronomy, anthropology, botany, ecology, ethnobotany, pathophysiology, phytosociology and taxonomy of several universities (national and international) and the Botanical Garden of Brazil, including undergraduate and graduate students, in 4 phases [10]. This participatory ethnobotany approach was implemented with the support of the local communities, including those who resided in these area even before the creation of the integral protection area in the Park, to support actions and generate integrated knowledge to make sustainable management plans, for better use of local plant resources.

The first phase began in March 2015, with a workshop organized by the managers of the Picinguaba Center of the Serra do Mar State Park, Ubatuba, SP, Brazil, where *Quilombolas* communities participated. During this event, participants identified a clear need for managers to support projects related to local biodiversity and social and cultural aspects, including economic alternatives for residents. Therefore, throughout the year, five meetings were held involving members of the two communities (QF and QC) and the research team, to develop collaborative research with objectives that would be of common interest.

### Collection of ethnobotanical and ethnoecological data (January 2016 to May 2018)

This study is part of the second phase of the project in which some members of the CQ and QF communities and university researchers co-developed project goals and methodologies, from the conception, sampling, collection and analysis of data [10, 19]. Meetings were held with the communities involved to co-define the objectives and activities of the study and community members were trained in data collection techniques including structured interview techniques [20], to document sociocultural data related to local knowledge (common name of the plant, part used, type of use, method of preparation, link between the collection of plants and the moon phase, possible restrictions to collection and collection instructions related to gender) and mainly herbal medicines (parts of prescribed plants, quantity and method of preparation, route of administration, time of use and possible contraindications). For the selection of the interviewees the 5 community collaborators invited all the 21 residents on the criteria of “being an expert in at least one of the following categories: medicinal, food / spices, civil construction, shipbuilding, handicrafts, combustion, others, hygiene / cosmetics, hunting, technology, dyeing and recreational [10, 21, 22]; 19 of them agreed to be part of the study. After obtaining the data on local knowledge, the community collaborators collected the specimens of species mentioned, which were identified and deposited in the Herbariums: Municipality of São Paulo (PMSP) and Instituto Florestal (SPSF).

During 178 days of fieldwork (see photos—bit.do/cee4, bit.do/cee5 and bit.do/cee6), 19 community members were interviewed by 5 community collaborators. In the QF 8 residents participated in the research, 5 women (62.5%) and 3 men (37.5%) with ages varying from 43 to 81 years old. All had incomplete elementary education, except one who has not studied. Occupations included artisans, farmers / farmers and one of the interviewees is a cook and works in the family restaurant. The 8 QF respondents generated a list of 92 plants. In the QC, 11 residents participated in the research, 2 women (18%) and 9 men (82%) aged between 35 and 65 years. All had incomplete elementary education, and worked as fishermen, cooks, farmers, bricklayers, and 6 of them live off the handicrafts they produce. The 11 QC respondents generated a list of 199 plants. This information was published in Yazbek [21] and Sauini [22]. Only 11.3% of the species were registered in both Quilombos. The categories dyes and foods / spices stand out for having the most common species in both communities, with 25% and 18.2%, respectively (Table 1).

**Table 1:** Number and percentage of plant species belonging to the 12 ethnobotanical categories reported by 11 interviewees of *Quilombo do Cambury* (*QC*) and eight of *Quilombo da Fazenda (QF)*. The species indicated in each quilombo total 199 (*QC*) and 92 (*QF*). As the same species may belong to more than one ethnobotanical category, they total 323 and 314 species, respectively.

Ethnobotanical categories	N° species cited in *QC*	N° species cited in *QF*	Total species cited in *quilombos*	N° and (%) species coincident in both *quilombos*
1.medicines	90	157	247	29 (11,7%)
2.food/spices	71	72	143	26 (18,2%)
3.construction	44	33	77	8 (11,1%)
4.shipbuilding	41	5	46	2 (4,3%)
5.handicraft	30	15	45	4 (8,9%)
6.tecnology	5	11	16	-
7.combustion	18	6	24	2 (8,3%)
8.hunting	5	4	9	-
9.tincture	2	2	4	1 (25%)
10.cosmetic	6	4	10	-
11.recreative	1	-	1	-
12.others	10	5	15	-
**Total**	**323**	**314**	**637**	**72 (11,3%)**

After the ethnobotanical and ethnoecological information was recorded, we carried out ecological studies (see below). The goal was to combine both sets of data to identify priority species for the development of sustainable use plans. Serra do Mar Park managers require these plans to allow residents to extract and market these plants in the form of crafts and others. This was one of the requests of the residents of these Quilombos and it can assist them in generating income, along with other activities they already perform with tourists.

### Sampling of phytosociological data (January 2017 to May 2018)

Quantitative studies on vegetation structure were performed by phytosociological method to characterize the forest used by *Quilombolas* and to provide data on species density.

Maps derived from aerial images were contextualized and presented to community members, who were then asked to identify areas commonly used for the collection of plant resources. Six areas were identified, two of which were selected in QC (A1: 523.502E and 7.416.881S; A2: 523.764E and 7.416.768S) and two in QF (A3: 516.970E and 7.419.302S; A4: 516.397E and 7.419 .005S), as those areas most used their collection. Therefore, in a later phase, transections were carried out for sampling and data collection in the respective areas [23].

**Fig 2:**
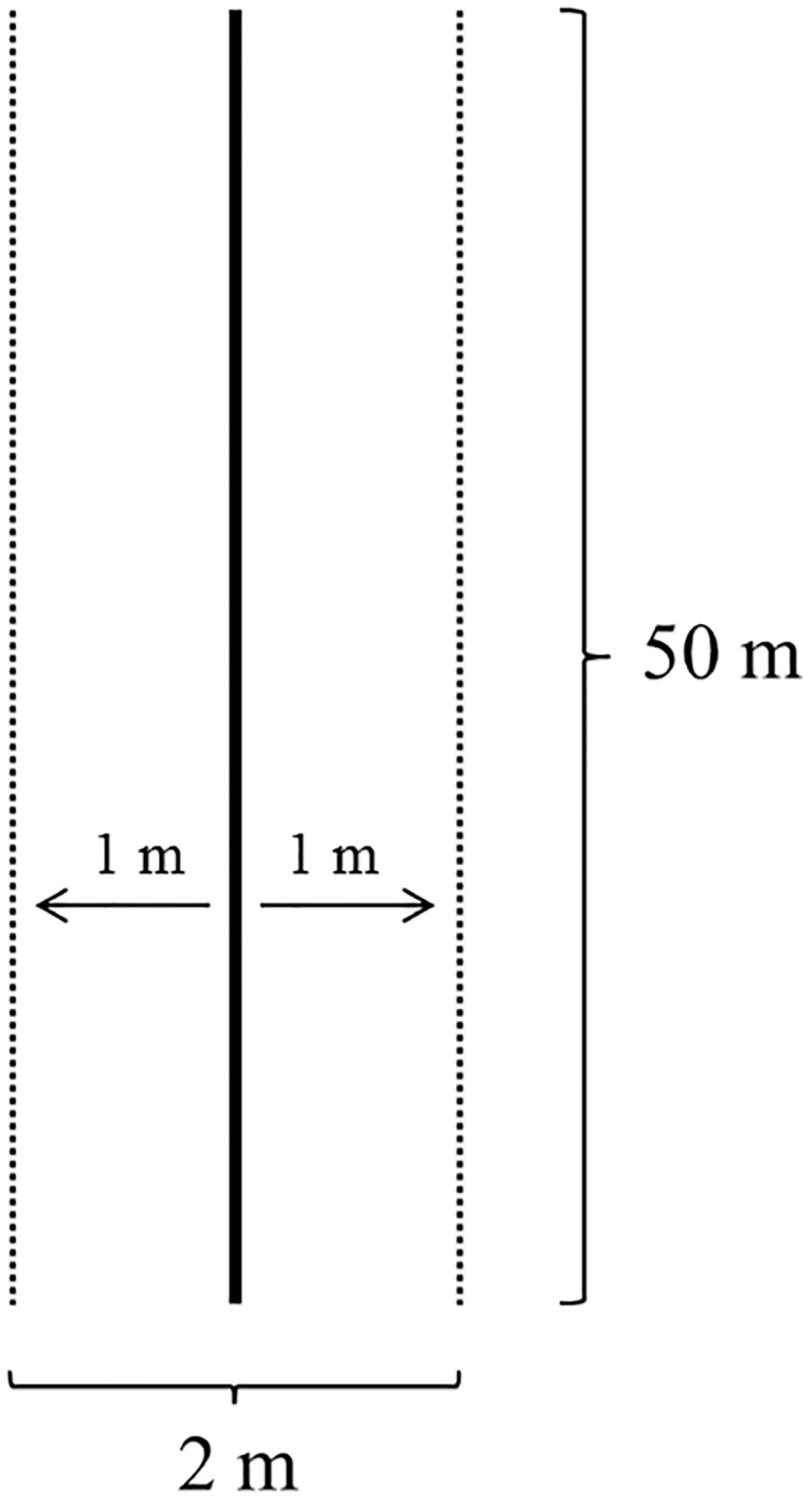
2m x 50m transection drawing (adapted from Gentry [24]) to identify the abundance of each species.

To identify the abundance of each species, ten 50x2m transects (adapted from Gentry [24]) were established, totaling 0.1 hectare in each *Quilombola* community (Fig 2). Trees, shrubs and tree ferns, with DBH (diameter at breast height or 1.30 m from the ground) equal to or above 4.8 cm., were sampled according Joly and collaborators [25]. For each individual, DBH, height and local name were noted. Fertile or vegetative samples were collected for later identification through pertinent bibliography and comparison with materials deposited in the PMSP and SP herbariums, adopting APG III [26]. The sampling effort was visualized using rarefaction curves for the sampling of each area from 100 randomizations, using EstimateS software [27], with the Jackknife-1 estimator. The number of individuals per species found in the transects was used to calculate relative density (see below).

The current conservation status of all species sampled was then determined from official threatened species lists such as: National Center for Conservation of Flora [28], Ministry of the Environment [29], Secretariat of Environment of São Paulo [30] and International Union for Conservation of Nature [31].

### Conservation priority analysis

To identify the degree of risk of collection of each species, we used the Conservation Priority Index (CPI) [5, 14, 32–35]. For all native species recorded from the transects, we carried out a bibliographic search to obtain the current state of conservation of these in Flora brasiliensis [36] and in the manual "Atlantic Forest Plants" [37].

The Conservation Priority Index was scored according to Table 2 and calculated using the following formula:

ConservationPriorityIndex:CPI=0,5(B)+0,5(RU)

where:

B = biological value
RU = risk of use

and:

B = Rd x 10 Rd = (N / ni) x100
Rd = relative density
N = individuals of species x
ni = individuals of all sampled species
RU = 0.5 (C) + 0.5 (U) x 10
C = Collection risk based on the botanical part collected
U = Value over use. This is determined by the highest value between L and Div

**Table 2:** Scoring criteria used to determine priority species for conservation [5], where: Rd—relative density; C—collection risk based on the botanical part collected; L—use location based on the reference frequency; Div—diversity or plurality of use assigned to the species.

Criteria		Score
**Rd**	Occurrence between 0 and 1 or very low.	10
	Occurrence between 1.1 and 3.5, or low.	7
	Occurrence between 3.6 and 7, or medium.	4
	Occurrence above 7 or moderate or high.	1
**C**	Removal of specimen from offspring, excluding possibility of perpetuation of the species.	10
	Removal of perennial structures without death, but actively influencing vegetative or flowering growth and perpetuation of species.	7
	Removal of permanent aerial parts without death only influences vegetative growth and energy production.	4
	The removal of transient aerial parts without direct influence on the life cycle of species.	1
**L**	Above 20%, its use is considered high.	10
	Between 10 and 20%, its use is considered moderately high.	7
	Up to 10%, its use is considered moderately low.	4
	Only mentioned in interviews.	1
**Div**	For each use, add 1.42 to the Div value.—Considering (7) different use categories.	Maximum 10

The species were then classified into three groups [33, 38]:

Category 1 (species with a score ≥ 85): at risk of extinction at the site and therefore of conservation priority; in need of a sustainable use management plan;
Category 2 (species with a score between 85 and 60): can likely tolerate moderate levels of collection;
Category 3 (species with score ≤ 60): suitable for continued collection.

### CPI based on successional categories

Finally, we then further divided species based on their successional categories. Although the CPI is recognized as the most efficient index to identify rare and impacted species in relation to the local vegetation [39], it doesn’t include species’ successional category, which may be relevant to conservation decisions. Therefore we classified species into three subdivisions based on local information as well as in other areas of the Atlantic Forest [40] as follows:

Subdivision A: includes old growth species (climax) and late secondary species found in more conserved forests;
Subdivision B: composed of early secondary species, uncommon in conserved areas, but more numerous in clearing areas and secondary forests;
Subdivision C: includes pioneer species—occurring in clearings, forest edges and degraded areas.

To classify species we used the works the works of Gandolfi and collaborators [41], Catharino and collaborators [42], and Barretto, Catharino [43]. We considered pioneer species as those with a short life cycle, fast growth and requiring high light for establishment and reproduction [43]. Early secondary species were considered to be fast-growing species with longer life cycles than the pioneer who show light-dependence but tolerate some shade. Late secondary species include long-lived species with shade-tolerant juveniles, these are generally slow-growing species typical of the mature canopy [43]. The ombrophilous category includes species that complete their entire life cycle in the shade of other trees, in the understory [42]. Plants considered as “conferatum”, undetermined or identified only at the genus level were grouped in the “unclassified” category.

## Results

**Fig 3:**
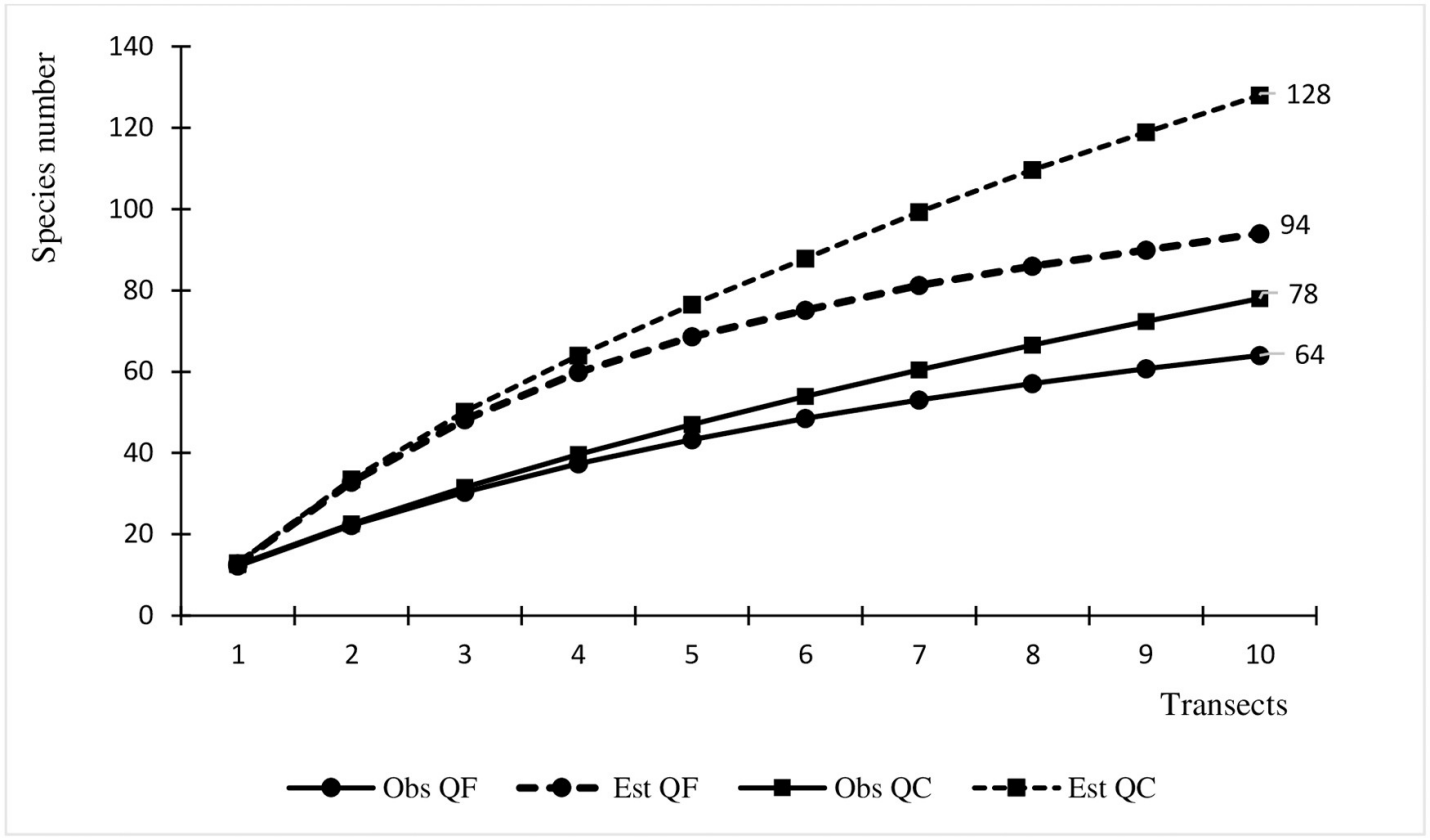
Graph of rarefaction curve with the number of species in relation to transects performed for the two study areas (QC and QF), with both observed (Obs) values and those estimated (Est) by Jackknife 1 using EstimateS software.

Fig 3 shows the rarefaction curve for the two study areas, with both observed values and those estimated with Jackknife 1 for QC (78–128) and QF (64–94), respectively.

Based on the combined ethnoecological data and the vegetation surveys, we assessed the conservation priority index for 113 species in 40 botanical families (Table 3).

**Table 3:** List of native forest species from ethnobotanical collection in *Quilombo do Cambury* (QC) and *Quilombo da Fazenda* (QF) communities in alphabetical order of family, containing information on species, common name, relative density, biological value, risk of use, Conservation Priority Index (CPI), risk categories (category 1—species with a score ≥ 85; category 2—species with a score between 85 and 60; category 3—species with score ≤ 60), Subdivision as to the criteria of natural occurrence (A—includes late and climatic secondary species found in more conserved forests; B—composed of early secondary species, uncommon in conserved areas, but more numerous in clearing areas and secondary forests; C—includes pioneer species—occurring in clearings, forest edges and degraded areas; NC—no classification) and successional category (PI—pioneer; IS—initial secondary; LS—late secondary; UM—umbrophilous; NC—no classification).

Family	Species	Common Name		Relative Density		Biological Value		Risk of Use		IPC		Risk Categories		Subdivision		Successional Category
		QC	QF	QC	QF	QC	QF	QC	QF	QC	QF	QC	QF	QC	QF	
Anacardiaceae	*Schinus terebinthifolius*		Aroeira		10		100		40		70		2		C	PI
Annonaceae	*Annona dolabripetala*	Araticum	São-roque	10	10	100	100	100	40	100	70	1	2	A	A	LS
Annonaceae	*Annona montana*	Graviola		10		100		25		62,5		2		A		LS
Annonaceae	*Guatteria australis*		Astro-de-fisga		10		100		85		92,5		1		A	LS
Annonaceae	*Xylopia brasiliensis*	Canafista		10		100		85		92,5		1		B		IS
Apocynaceae	*Malouetia cestroides*	Guairana		10		100		85		92,5		1		C		PI
Apocynaceae	*Tabernaemontana laeta*		Guarana		10		100		55		77,5		2		C	PI
Araliaceae	*Schfflera* cf. *Angustissima*	Imbirotó		7		70		70		70		3		NC		NC
Arecaceae	*Astrocaryum aculeatissimum*	Brejaúba			10		100		85		92,5		1		A	UM
Arecaceae	*Euterpe edulis*	Juçara	Juçara	1	1	10	10	25	100	17,5	55	3	3	A	A	UM
Arecaceae	*Geonoma* sp.	Urecanga		10		100		85		92,5		1		NC		NC
Arecaceae	*Syagrus pseudococos*		Patiuava		10		100		100		100		1		B	IS
Asteraceae	*Vernonanthura beyrichii*	Cambará		10		100		55		77,5		2		C		PI
Bignoniaceae	*Cybistax antisyphilitica*	Cinco-folhas		10		100		55		77,5		2		A		LS
Bignoniaceae	*Handroanthus albus*	Ipe-amarelo	Ipê-amarelo	10	10	100	100	85	85	92,5	92,5	1	1	A	A	LS
Bignoniaceae	*Handroanthus impetiginosus*		Ipê-roxo		10		100		85		92,5		1		A	LS
Bignoniaceae	*Jacaranda puberula*	Caroba-branca	Carobinha	10	10	100	100	55	55	77,5	77,5	2	2	B	B	IS
Bignoniaceae	*Tabebuia cassinoides*		Caxeta		10		100		85		92,5		1		C	PI
Boraginaceae	*Cordia sellowiana*			7		70		100		85		1		B		IS
Boraginaceae	*Cordia* sp. 1	Louro	Louro	10	10	100	100	70	85	85	92,5	1	1	NC	NC	NC
Boraginaceae	*Cordia* sp. 2	Louro-pardo		10		100		100		100		1		NC		NC
Cannabaceae	*Trema micrantha*	Candiúva		10		100		100		100		1		C		PI
Caricaceae	*Jacaratia spinosa*	Mamão-do-mato		10		100		25		62,5		2		B		IS
Celastraceae	*Monteverdia ardisiifolia*	Guaracipó		10		100		70		85		1		A		UM
Chloranthaceae	*Hedyosmum brasiliense*	Congonha		10		100		70		85		1		B		IS
Chrysobalanaceae	*Licania* sp.	Milho-torrado		10		100		70		85		1		NC		NC
Clusiaceae	*Clusia criuva* subsp. P*arviflora*	Figueira-braçadeira		10		100		85		92,5		1		C		PI
Clusiaceae	*Garcinia gardneriana*	Bacupari	Bacupari	10	7	100	70	85	85	92,5	77,5	1	2	A	A	LS
Combretaceae	*Buchenavia kleinii*	Angelim		10		100		70		85		1		A		LS
Erythroxylaceae	*Erythroxylum pulchrum*		Guará-cipó		10		100		85		92,5		1		A	LS
Euphorbiaceae	*Actinostemon verticillatus*	Sucanga		10		100		100		100		1		A		LS
Euphorbiaceae	*Mabea piriri*	Canudo-de-pito	Canudo-de-pito	1	4	10	40	100	85	55	62,5	3	3	C	C	PI
Euphorbiaceae	*Maprounea* sp.	Espera		10		100		70		85		1		NC		NC
Euphorbiaceae	*Tetrorchidium* sp.	Bapeva		10		100		70		85		1		NC		NC
Fabaceae	*Albizia pedicellaris*		Timbuíba		10		100		85		92,5		1		C	PI
Fabaceae	*Albizia* sp.	Timbuíva		10		100		70		85		1		NC		NC
Fabaceae	*Andira fraxinifolia*	Sucupira		10		100		70		85		1		B		IS
Fabaceae	cf. *Dalbergia frutescens*	Braço-forte		10		100		70		85		1		NC		NC
Fabaceae	cf. *Hymenolobium janeirense*	Guacuí		10		100		85		92,5		1		NC		NC
Fabaceae	cf. *Pterocarpus rohrii*		Guaricica-amarela		10		100		85		92,5		1		NC	NC
Fabaceae	cf. *Swartzia oblata*	Jatobá		10		100		55		77,5		2		NC		NC
Fabaceae	*Hymenaea altíssima*	Jatobá	Jatobá	10	10	100	100	100	100	100	100	1	1	A	A	LS
Fabaceae	*Inga* cf. *lenticellata*	Ingá-ferro		10		100		70		85		1		NC		NC
Fabaceae	*Inga edulis*		Ingá-de-metro		10		100		55		77,5		2		B	IS
Fabaceae	*Inga marginata*	Ingá-feijão	Ingá-feijão	10	10	100	100	25	40	62,5	70	2	2	B	B	IS
Fabaceae	*Inga* sp.			10		100		70		85		1		NC		NC
Fabaceae	*Myrocarpus frondosus*		Cabreúva		10		100		85		92,5		1		A	LS
Fabaceae	*Piptadenia gonoacantha*		Caniveteiro		10		100		85		92,5		1		C	PI
Fabaceae	*Pseudopiptadenia leptostachya*	Cobi		10		100		70		85		1		B		IS
Fabaceae	*Schizolobium parayba*		Guapuruvu		10		100		85		92,5		1		C	PI
Fabaceae	*Swartzia oblata*	Barbatimão	Barbatimão	10	10	100	100	70	100	85	100	1	1	A	A	LS
Fabaceae	*Swartzia simplex* var. *grandiflora*	Laranjeira-do-mato	Canela-prego	7	10	70	100	70	85	70	92,5	3	1	A	A	LS
Fabaceae	*Tachigali paratyensis*	Ingá-flecha		10		100		85		92,5		1		A		LS
Fabaceae	*Tachigali* sp. 1	Ingá-amarelo		10		100		85		92,5		1		NC		NC
Fabaceae	*Tachigali* sp. 2	Ingá-flecha		10		100		85		92,5		1		NC		NC
Fabaceae	*Tachigali* sp. 3	Ingá-fedido		10		100		85		92,5		1		NC		NC
Lacistemaceae	*Lacistema lucidum*	Tatuzinho	Borrachudo	7	10	70	100	85	85	77,5	92,5	2	1	B	B	IS
Lamiaceae	*Aegiphila integrifolia*	Cajuja	Cajuja	10	10	100	100	70	85	85	92,5	1	1	C	C	PI
Lamiaceae	*Vitex polygama*	Tarumã		10		100		70		85		1		B		IS
Lauraceae	*Aniba* sp.	Canela-do-mato		10		100		70		85		1		NC		NC
Lauraceae	*Cryptocarya* cf. *mandioccana*	Nóz-moscada	Nóz-moscada	10	10	100	100	25	55	62,5	77,5	2	2	NC	A	LS
Lauraceae	*Cryptocarya saligna*		Canela-sassafraize		10		100		100		100		1		A	LS
Lauraceae	*Nectandra oppositifolia*	Canela-do-mato		10		100		85		92,5		1		B		IS
Lecythidaceae	*Cariniana estrellensis*	Jequitibá		10		100		85		92,5		1		A		LS
Malvaceae	*Eriotheca pentaphylla*	Imbiruçu		10		100		70		85		1		A		LS
Melastomataceae	*Huberia ovalifolia*	Tinteiro	Tinteiro	10	10	100	100	70	85	85	92,5	1	1	A	A	UM
Melastomataceae	*Miconia cinnamomifolia*	Jacatirão	Jacatirão	10	7	100	70	70	85	85	77,5	1	2	B	B	IS
Melastomataceae	*Miconia dodecandra*	Pixirica		10		100		25		62,5		2		B		IS
Melastomataceae	*Miconia prasina*	Pixiricão		10		100		25		62,5		2		B		IS
Melastomataceae	*Tibouchina pulchra*	Quaresmeira		7		70		100		85		1		C		PI
Meliaceae	*Cabralea canjerana*	Ingá-cajarana		10		100		85		92,5		1		A		LS
Meliaceae	*Cedrela* cf. *odorata*	Cedro		10		100		70		85		1		NC		NC
Meliaceae	*Cedrela fissilis*	Cedro-rosa	Cedro-rosa	10	10	100	100	100	100	100	100	1	1	A	A	LS
Meliaceae	*Guarea macrophylla*	Café-do-mato	Café-do-mato	10	7	100	70	70	7	85	38,5	1	3	A	A	UM
Moraceae	*Brosimum guianense*	Guaricica-da-vermelha		10		100		85		92,5		1		A		LS
Moraceae	*Ficus adhatodifolia*	Figueira-branca	Figueira	10	10	100	100	70	100	85	100	1	1	A	A	LS
Moraceae	*Ficus gomelleira*	Figueira-parda		10		100		85		92,5		1		A		LS
Moraceae	*Sorocea cf*. *guilleminiana*	Espineira-santa	Garapinha	10	10	100	100	85	85	92,5	92,5	1	1	NC	NC	NC
Myristicaceae	*Virola bicuhyba*	Bicuiba		10		100		100		100		1		A		LS
Myrtaceae	*Campomanesia phaea*	Cambuci	Cambuci		10		100		55		77,5		2		A	UM
Myrtaceae	*Eugenia astringens*		Araçarana		10		100		85		92,5		1		A	UM
Myrtaceae	*Eugenia brasiliensis*		Grumixama		10		100		40		70		2		A	UM
Myrtaceae	*Eugenia* cf. *multicostata*	Carambola-do-mato		10		100		40		70		2		NC		NC
Myrtaceae	*Eugenia* cf. *stipitata*		Araça-do-norte		10		100		100		100		1		NC	NC
Myrtaceae	*Eugenia sulcata*	Pitanga-do-mato		10		100		25		62,5		2		A		UM
Myrtaceae	*Eugenia uniflora*	Pitanga	Pitanga	10	10	100	100	55	70	77,5	85	2	1	A	A	UM
Myrtaceae	*Myrcia neoriedeliana*		Cambucá-do-mato		10		100		40		70		2		A	UM
Myrtaceae	*Myrcia spectabilis*	Arueira	Arco-de-peneira	10	10	100	100	70	85	85	92,5	1	1	B	B	IS
Myrtaceae	*Plinia edulis*	Cambucá	Cambucá	10	10	100	100	100	40	100	70	1	2	A	A	UM
Myrtaceae	*Plinia* sp.	Jaboticaba		10		100		25		62,5		2		NC		NC
Myrtaceae	*Psidium cattleianum*	Aracá	Araçá	10	10	100	100	85	70	92,5	85	1	1	B	B	IS
Myrtaceae	*Psidium guajava*	Goiaba	Goiaba	10	10	100	100	70	100	85	100	1	1	B	B	IS
Nyctaginaceae	*Guapira nitida*			10		100		55		77,5		2		A		UM
Peraceae	*Pera glabrata*	Chile	Chile	10	10	100	100	85	85	92,5	92,5	1	1	B	B	IS
Phyllanthaceae	*Hyeronima alchorneoides*	Aricurana	Aricurana	7	7	70	70	70	100	70	85	3	1	B	B	IS
Phytolaccaceae	*Gallesia integrifolia*		Pau d’alho		10		100		85		92,5		1		A	LS
Primulaceae	*Myrsine coriacea*	Capororoca	Capororoca	7	10	70	100	85	85	77,5	92,5	2	1	C	C	PI
Primulaceae	*Stylogyne lhotzkyana*	Sapopema		10		100		70		85		1		A		UM
Rubiaceae	*Bathysa mendoncaei*	Sapopema			10		100		85		92,5		1		A	UM
Rubiaceae	cf. *Bathysa*		Aribarrosa		10		100		85		92,5		1		NC	NC
Rubiaceae	*Faramea hymenocalyx*		Catinga-de-porca		10		100		85		92,5		1		A	UM
Rubiaceae	*Rustia formosa*	Manduberana	Guacá	10	10	100	100	70	50	85	75	1	2	A	A	UM
Rutaceae	*Dictyoloma vandellianum*			10		100		25		62,5		2		C		PI
Rutaceae	*Zanthoxylum rhoifolium*	Mamica-de-moça	Mamica-de-porca	10	10	100	100	70	100	85	100	1	1	B	B	IS
Sapindaceae	*Cupania oblongifolia*	Cubatã	Cubatã	10	7	100	70	100	85	100	77,5	1	2	A	A	LS
Sapotaceae	*Ecclinusa ramiflora*	Guacá		10		100		100		100		1		A		LS
Sapotaceae	*Pouteria caimito*		Guapeva		10		100		85		92,5		1		A	LS
Sapotaceae	*Pouteria sp*. *2*	Guacuáuçu		10		100		85		92,5		1		NC		NC
Solanaceae	*Solanum pseudoquina*	Piloteira	Piloteira	10	10	100	100	100	85	100	92,5	1	1	C	C	PI
Urticaceae	*Cecriopia glaziovii*	Embaúba-vermelha	Embaúba	10	10	100	100	100	100	100	100	1	1	C	C	PI
Urticaceae	*Cecropia pachystachya*	Embaúba-branca		10		100		40		70		2		C		PI
Urticaceae	*Pourouma guianensis*	Baubu		10		100		85		92,5		1		B		IS
Verbenaceae	*Citharexylum myrianthum*	Tarumã		10		100		70		85		1		B		IS

In the QC, 214 individuals were inventoried in the transects, distributed in 88 species from 37 families. The most abundant species were Palmito-jussara (*Euterpe edulis*) and Canudo-de-pito (*Mabea piriri*), representing 66 (30.8%) and 21 (9.8%) individuals, respectively.

In the QF, 158 individuals were sampled, distributed in 58 species from 28 families. The most abundant species were Palmito-jussara (*Euterpe edulis*) and Canudo-de-pito (*Mabea piriri*), representing 17 (10.7%) and 6 (3.7%) individuals, respectively.

In terms of the successional stage of the inventoried species, there are 18 pioneers (PI), 24 initial secondary (IS), 29 late secondary (LS), 17 umbrophilous (UM) and 25 without classification (NS).

Of the native species analyzed in relation to the Conservation Priority Index categories, in QC, 64 are in Category 1 (72.7% of the total sampled species), 12 of which are most relevant in that they have the maximum CPI value (100). In QF, 40 species are in Category 1 (68.9% of the total sampled species), 10 of which are the most relevant in terms of having the maximum CPI value (100).

In terms of conservation status in global conservation lists utilized, there were 11 species in the categories: "least concern" (LC), "almost threatened" (NT), "vulnerable" (VU) and "endangered" (EN) (Table 4).

**Table 4:** Local species listed of conservation concern and status (LC—least concern; NT—almost threatened; VU—vulnerable; EN—endangered).

	Specie	Conservation Status
**QC**	*Buchenavia kleinii*	LC
	*Guatteria australis*	LC
	*Handroanthus albus*	LC
	*Cedrela fissilis*	VU
	*Plinia edulis*	VU
	*Virola bicuhyba*	EN
**QF**	*Astrocaryum aculeatissimum*	LC
	*Handroanthus albus*	LC
	*Erythroxylum pulchrum*	LC
	*Myrocarpus frondosus*	LC
	*Swartzia simplex* var. *grandiflora*	LC
	*Handroanthus impetiginosus*	NT
	*Cedrela fissilis*	VU

## Discussion

**Fig 4:**
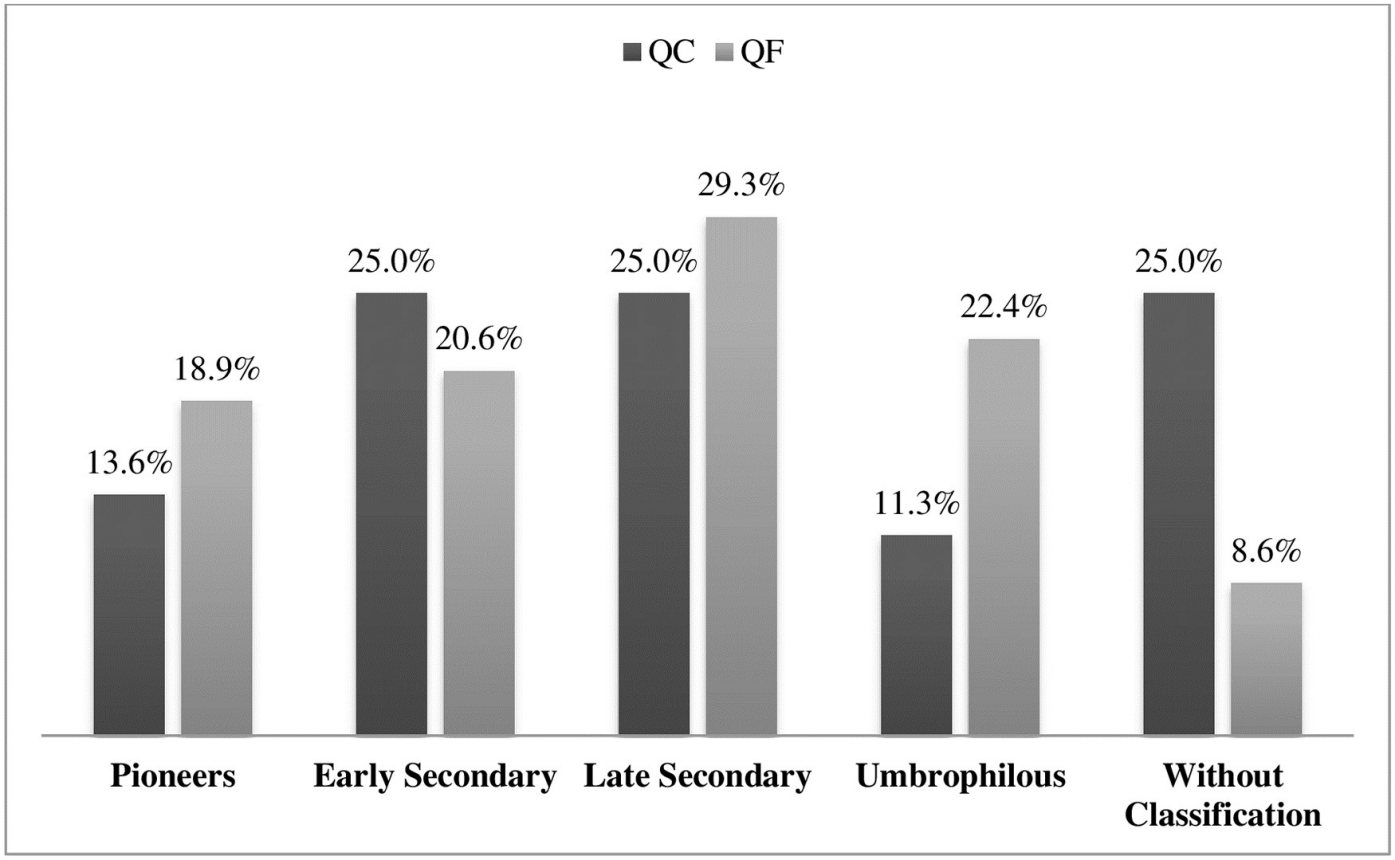
Percentage of species occurring in the present sample as to their successional categories in QC and QF.

The rarefaction curves both communities start to level off indicating sufficient sampling. The two stretches of forest sampled in the CQ are close to the Cambury beach access road and have had anthropogenic interventions in the past, such as shallow or selective botany exploration. In that region, there is a history of land use for agriculture [44], especially monocultures, initially sugarcane and then coffee [45]. The prevalence of umbrophilous species in the QF indicate that the access areas for collecting raw material are better preserved than in the QC (Fig 4).

About 70% of species in both communities fell into the highest threat category (Category 1). These values are higher than those recorded in other studies. In *Quilombola* communities in the Atlantic Forest. Crepaldi, Peixoto [14] documented only 10.76% of the sampled species in the Cachoeira do Retiro Community (Espírito Santo) as Category 1. Conde and collaborators [5] documented 52% in the community of São Bento (Minas Gerais) and 56% in the community of São Sebastião da Boa Vista (Minas Gerais).

The high CPI values we recorded may be due in part to our sampling methodology, and demonstrate the importance of including successional category in this kind of analysis. For example, several common species from anthropogenic areas were classified in Category 1. This included Embauba-vermelha (*Cecropia glaziovii*), Cajuja (*Aegiphila integrifolia*) and Candiúva (*Trema micrantha*) in QC, and Capororoca (*Myrsine coriacea*), Caniveteiro (*Piptadenia gonoacantha*) and Guapuruvu (*Schizolobium parahyba*) in QF. All are pioneer species [43] and occur in clearings [46], forest edges and degraded areas. However, the areas identified by community members as the most important collection sites–and where the transects were therefore placed- were closed canopy areas (low light penetration). Therefore a low density of pioneer species is expected and the CPI values not fully representative.

Similarly, non-pioneer species included in Category 1 also included those found in low canopy cover forest environments and more associated with cleared environments and forest fragment borders. We also did not sample these habitats. In QC, this included Cedro-rosa (*Cedrella fissilis*), Cubatan (*Cupania oblongifolia*), Canafista (*Xylopia brasiliensis*) and Café-do-mato (*Guarea macrophylla*); and in QF Cedro-rosa (*Cedrella fissilis*) [44]. However, non-pioneer species included in Category 1 also included Guaracipó (*Maytenus ardisiaefolia*), Ingá-flecha (*Tachigali paratyensis*), Tinteiro (*Huberia ovalifolia*) and Figueira (*Ficus adhatodifolia*) in QC; and Guará-cipó (*Erythroxylum pulchrum*), Tinteiro (*Huberia ovalifolia*), Figueira (*Ficus adhatodifolia*) and Catinga-de-nut (*Faramea hymenocalyx*) in QF. These species are found in more conserved forests, and are recorded as naturally rare [43].

### Selection of priority species for the development of sustainable use management plans

To select priority species, we focused on late and umbrophilous secondary plants. There were 8 late and umbrophilous secondary species with the highest CPI values (of 100) in QC (*Annona dolabripetala*, *Actinostemon verticillatus*, *Hymenaea altíssima*, *Cedrela fissilis*, *Virola bicuhyba*, *Plinia edulis*, *Cupania oblongifolia*, *Ecclinusa ramiflora*) and 5 species (*Hymenaea altissima*, *Swartzia oblata*, *Cryptocarya saligna*, *Cedrela fissilis*, *Ficus adhatodifolia*) in QF. When overlaid with the species officially listed as threatened or endangered at the level country, there candidate species emerged: Bicuíba (*Virola bicyhyba*), Cambucá (*Plinia edulis*) and Cedro-rosa (*Cedrela fissilis*). These represent priority species for which to develop sustainable use plans–they are both ethnobotanically highly important and ecologically at risk locally. Is important would highlight a chose to overlay the national priorities with the local priorities. Species that are of very high local priority may not be a national priority, but they might be the most important to address locally. Sustainable use plans can help conserve the species while contributing to the quality of life of local populations [47]:

Priority 1—Endangered (EN)
  - *Virola bicuhyba* (QC used as fuel)*—*According to CNCFlora [48], a loss of more than 65% of *V*. *bicuhyba* cover was reported within its known extent of occurrence; a population reduction of more than 60% was found in the last three generations of the taxon (estimated at about 30 years), caused mainly by selective extraction and habitat conversion, which will continue to cause future decline if nothing will be done according to its conservation. For these reasons, the species *V*. *bicuhyba* is considered threatened with extinction, requiring the creation of protected areas to ensure its survival and the development of specific legislation that regulates and controls its use in an appropriate manner. This species is of great importance to the regional economy in various locations and its total restriction can cause impacts.
Priority 2—Vulnerable (VU)
  - *Plinia edulis* (QF used as food)*—*According to CNCFlora [49] *P*. *edulis* is a species with edible and widely appreciated fruits and is therefore highly cultivated. However, it is quite rare in nature, with a population estimate of about 10,000 adult individuals. It is found outside protected areas and is therefore expected to face a population reduction of more than 10% over the next 30 years, considering a generation time of about ten years. In addition, the species occurs in places under strong anthropogenic pressure that have suffered habitat loss greater than 80%. It is therefore assumed that there has been a population reduction of more than 30% in the last 30 years. Thus, the species was therefore categorized as Vulnerable (VU).
  - *Cedrela fissilis* (QC used for shipbuilding; QF used for construction, shipbuilding and medicine)—The species has historically been suffering from logging throughout its occurrence, which has led many of the subpopulations to extinction. In addition, most of its habitat has been completely degraded and converted into urban areas, pastures, plantations, among others. Due to these factors, it is suspected that *C*. *fissilis* has experienced a population decline of at least 30% over the last three generations, according to IUCN [50].

## Conclusion

Our methodology allowed us to identify three species to prioritize for the co-development of sustainable management plans: these species are of high importance to the local communities, and both locally and globally threatened. The development of sustainable management plans requires consideration of harvest methods that will allow for their long-term resilience [51] as well as of potential alternatives, such as the promotion of species in agroforestry programs and/or the development of alternative uses for the species, that together can ensure the maintenance of cultural traditions and quality of life while preserving wildlife and the nature.
